# The genome sequence of the Chestnut, *Conistra vaccinii *(Linnaeus, 1761)

**DOI:** 10.12688/wellcomeopenres.20346.1

**Published:** 2023-11-17

**Authors:** David C. Lees, Natural History Museum Genome Acquisition Lab, Darwin Tree of Life Barcoding collective, Wellcome Sanger Institute Tree of Life programme, Wellcome Sanger Institute Scientific Operations: DNA Pipelines collective, Tree of Life Core Informatics collective, Darwin Tree of Life Consortium

**Affiliations:** 1 Natural History Museum, London, England, UK

**Keywords:** Conistra vaccinii, the Chestnut, genome sequence, chromosomal, Lepidoptera

## Abstract

We present a genome assembly from an individual male *Conistra vaccinii* (the Chestnut; Arthropoda; None; Lepidoptera; Noctuidae). The genome sequence is 720.8 megabases in span. Most of the assembly is scaffolded into 31 chromosomal pseudomolecules, including the Z sex chromosome. The mitochondrial genome has also been assembled and is 15.44 kilobases in length. Gene annotation of this assembly on Ensembl identified 13,109 protein coding genes.

## Species taxonomy

Eukaryota; Metazoa; Eumetazoa; Bilateria; Protostomia; Ecdysozoa; Panarthropoda; Arthropoda; Mandibulata; Pancrustacea; Hexapoda; Insecta; Dicondylia; Pterygota; Neoptera; Endopterygota; Amphiesmenoptera; Lepidoptera; Glossata; Neolepidoptera; Heteroneura; Ditrysia; Obtectomera; Noctuoidea; Noctuidae; Cuculliinae; *Conistra*; *Conistra vaccinii* (Linnaeus, 1761) (NCBI:txid706635).

## Background

The Chestnut, *Conistra vaccinii* is a smallish to medium sized (ca. 33–36 mm wingspan, 14–15 mm forewing length) ( Bretherton *et al.*, 1983; McMannis & Fiedler, 2019) noctuid moth, usually with a black mark in the lower part of the reniform stigma of the forewing, colouration varying from plain reddish or chestnut brown to a pattern marbled with yellower brown or grey. It is distinguished from the very similar *C. ligula* (Esper, 1791) by the relatively unpointed forewing apex with also a more rounded termen. The adult emerges in the Autumn, flying between September and November and overwintering as an adult, reappearing in February to May ( Randle *et al.*, 2019). Like some other members of its genus, *C. vaccinii* awakes during hibernation to feed during the winter; by contrast, adults of *C. ligula* die off near the beginning of the new year. Feeding on fruit during the winter increases fecundity but not apparently longevity ( McMannis & Fiedler, 2019).

*Conistra vaccinii* is found most often in non-coniferous woodland, also scrub, heathland, hedgerows and gardens in the UK ( Waring *et al.*, 2017). The adult, emerging in September and October, is attracted to the flowers of ivy ( *Hedera helix* L.) and ripe blackberries and other fruits, feeding on *Salix* blossoms in the Spring, by which time it can lay fertile eggs ( Bretherton *et al.*, 1983). The eggs hatch within 11 to 14 days, and the larva feeds from late April to June on deciduous trees and shrubs such as blackthorn, hawthorn, birch and sweet chestnuts, sometimes later descending to feed on herbaceous plants ( Waring *et al.*, 2017). Full-grown, it is about 30 mm long and pupates in the ground in the loose cocoon ( Bretherton *et al.*, 1983), pupating about two months later ( Waring *et al.*, 2017).

The Chestnut is generally common and widespread in Britain and the Isle of Man and Channel Islands (sparsely recorded in Ireland) ( NBN Atlas Partnership, 2023), and in the Palaearctic as far east as Central Asia and as far south as the shores of the Mediterranean, but it is absent in northern Scandinavia and northern Russia ( GBIF Secretariat, 2023). Both abundance (+36%) and distribution (+41%) – notably in Scotland – have increased since 1970 ( Randle *et al.*, 2019).

*Conistra vaccinii* is currently placed in the noctuid tribe Xylenini, subtribe Xylenina. *Conistra vaccinii* has been treated as part of the subgenus *Conistra* in the checklist of related genera by Benedek *et al*. (2022). It would be interesting to use genomic data to explore its closest relatives. In a multigenomic study of noctuids using eight markers, Keegan *et al*. (2021) (20, Fig. 6) grouped *C. vaccinii* among Xylenini with *Litholomia napaea* (Morrison, 1874), *Omphaloscelis lunosa* (Haworth, 1909) as well as *Conistra rubiginea* ([Denis & Schiffermüller], 1775) + *Anathix ralla* (Grote & Robinson, 1868), thus the monophyly of *Conistra* is not certain. On BOLD there is a single DNA barcode cluster (BIN, BOLD:AAB7880) which *C. vaccinii* shares with *Conistra ligula* (Esper, 1871) with only a small divergence (from about 1%) and which it also shares with *C. alicia* Lajonquière, 1939 ( Huemer & Wieser, 2023).

## Genome sequence report

**Figure 1.**
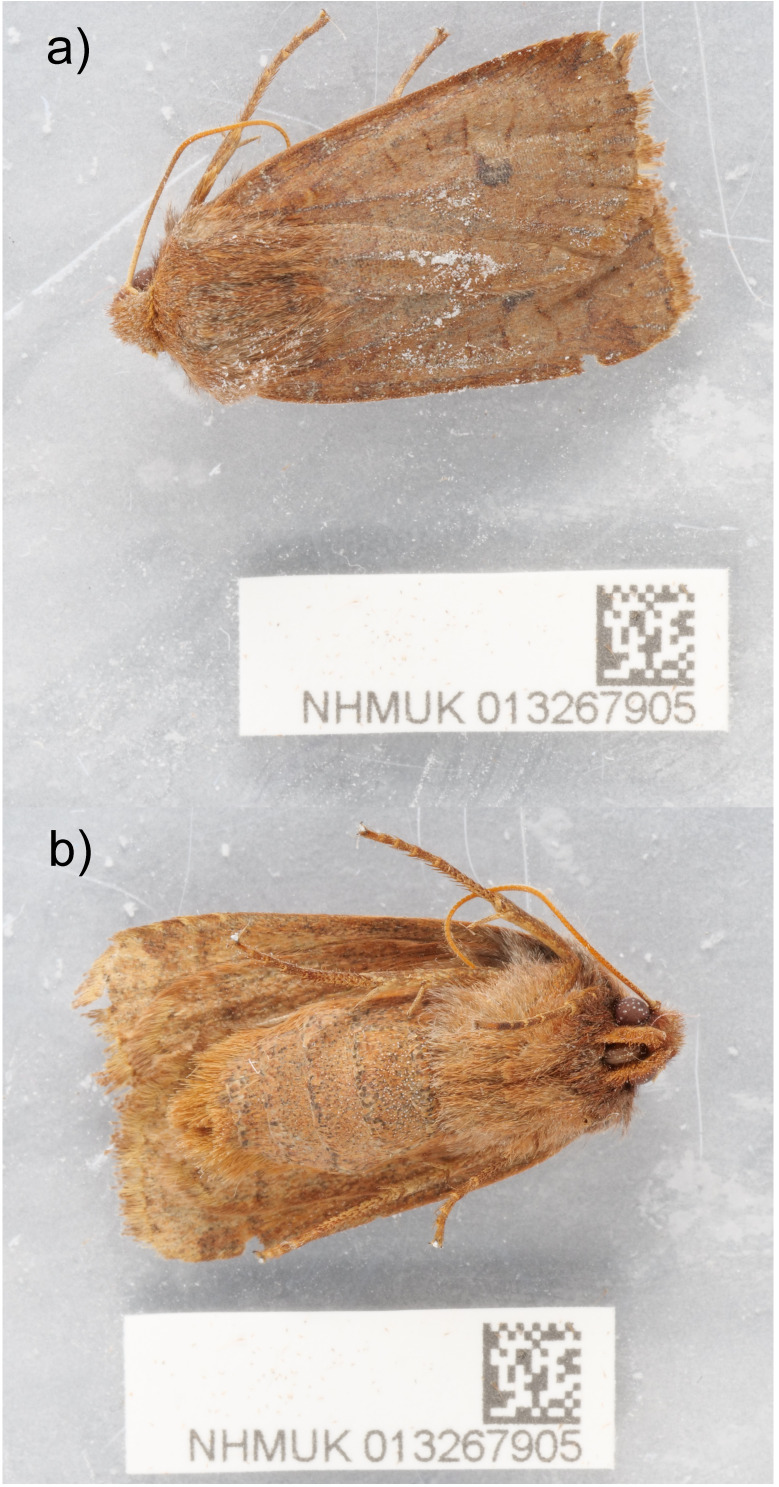
Photograph of the *Conistra vaccinii* (ilConVacc3) specimen used for genome sequencing.

The genome was sequenced from one male *Conistra vaccinii* ( Figure 1) collected from High Wycombe, Buckinghamshire, UK (51.63, –0.74). A total of 36-fold coverage in Pacific Biosciences single-molecule HiFi long reads was generated. Primary assembly contigs were scaffolded with chromosome conformation Hi-C data. Manual assembly curation corrected 9 missing joins or mis-joins and removed 4 haplotypic duplications, reducing the assembly length by 0.8%.

**Figure 2.**
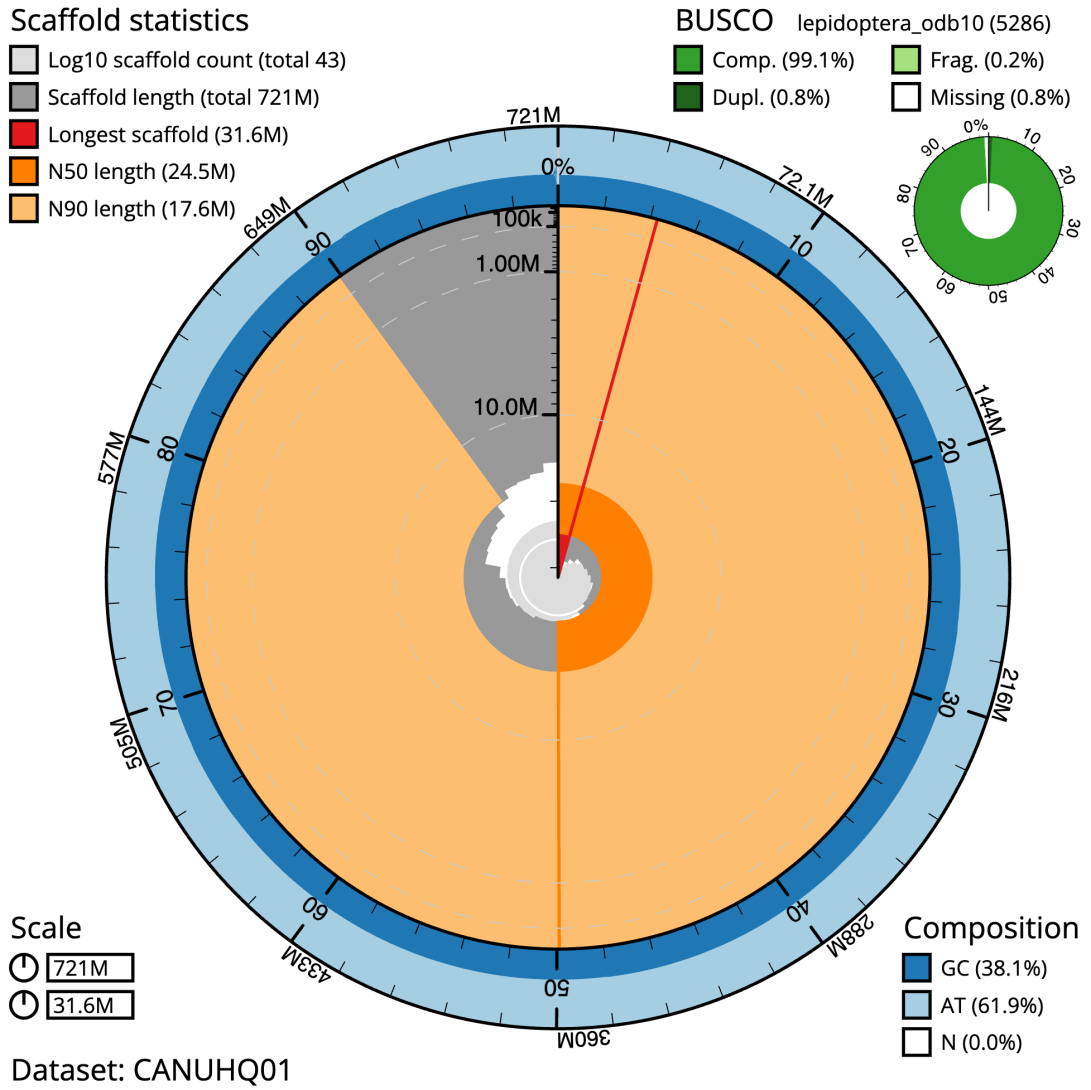
Genome assembly of *Conistra vaccinii*, ilConVacc3.1: metrics. The BlobToolKit Snailplot shows N50 metrics and BUSCO gene completeness. The main plot is divided into 1,000 size-ordered bins around the circumference with each bin representing 0.1% of the 720,850,966 bp assembly. The distribution of scaffold lengths is shown in dark grey with the plot radius scaled to the longest scaffold present in the assembly (31,631,061 bp, shown in red). Orange and pale-orange arcs show the N50 and N90 scaffold lengths (24,458,057 and 17,604,836 bp), respectively. The pale grey spiral shows the cumulative scaffold count on a log scale with white scale lines showing successive orders of magnitude. The blue and pale-blue area around the outside of the plot shows the distribution of GC, AT and N percentages in the same bins as the inner plot. A summary of complete, fragmented, duplicated and missing BUSCO genes in the lepidoptera_odb10 set is shown in the top right. An interactive version of this figure is available at https://blobtoolkit.genomehubs.org/view/Conistra%20vaccinii/dataset/CANUHQ01/snail.

**Figure 3.**
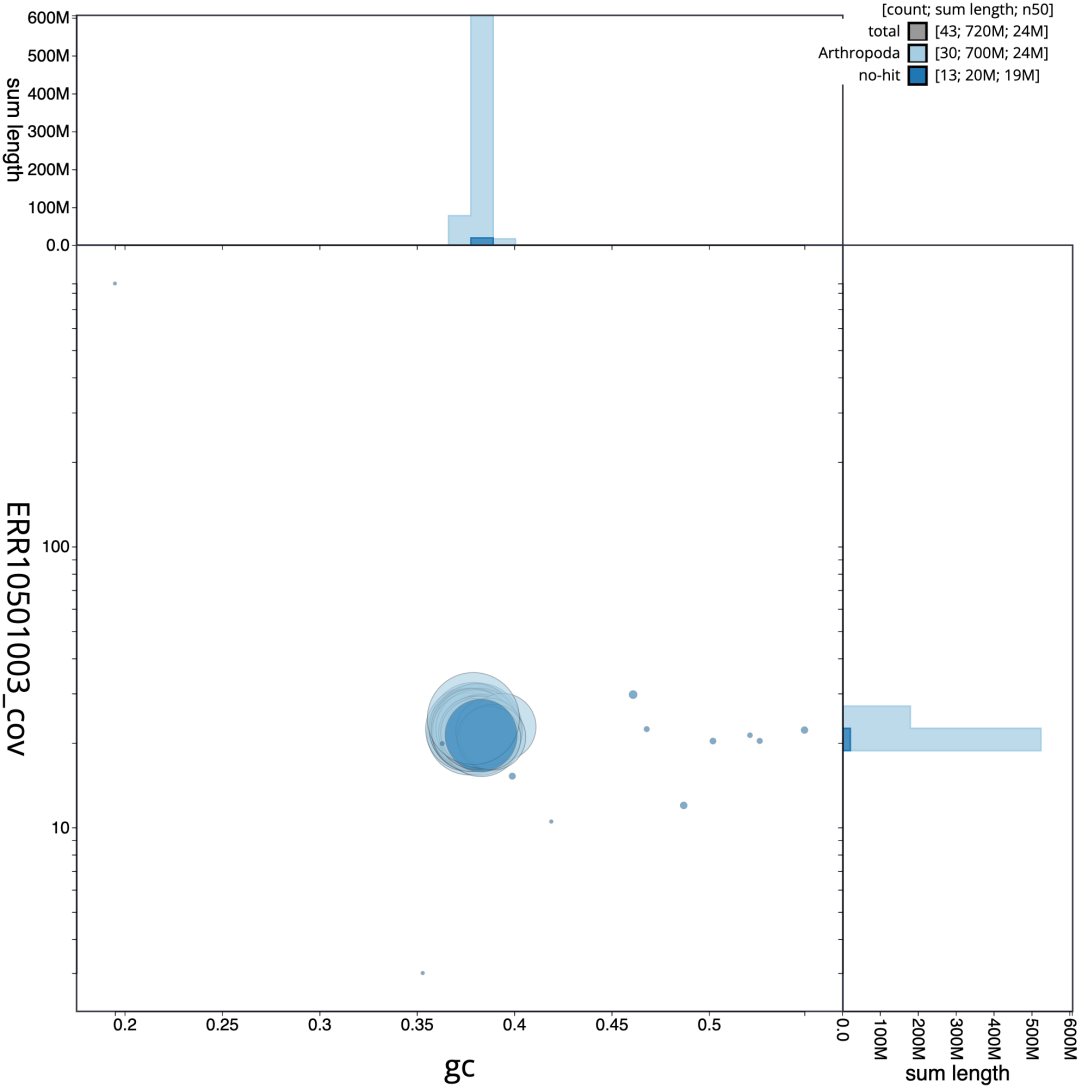
Genome assembly of *Conistra vaccinii*, ilConVacc3.1: BlobToolKit GC-coverage plot. Scaffolds are coloured by phylum. Circles are sized in proportion to scaffold length. Histograms show the distribution of scaffold length sum along each axis. An interactive version of this figure is available at https://blobtoolkit.genomehubs.org/view/Conistra%20vaccinii/dataset/CANUHQ01/blob.

**Figure 4.**
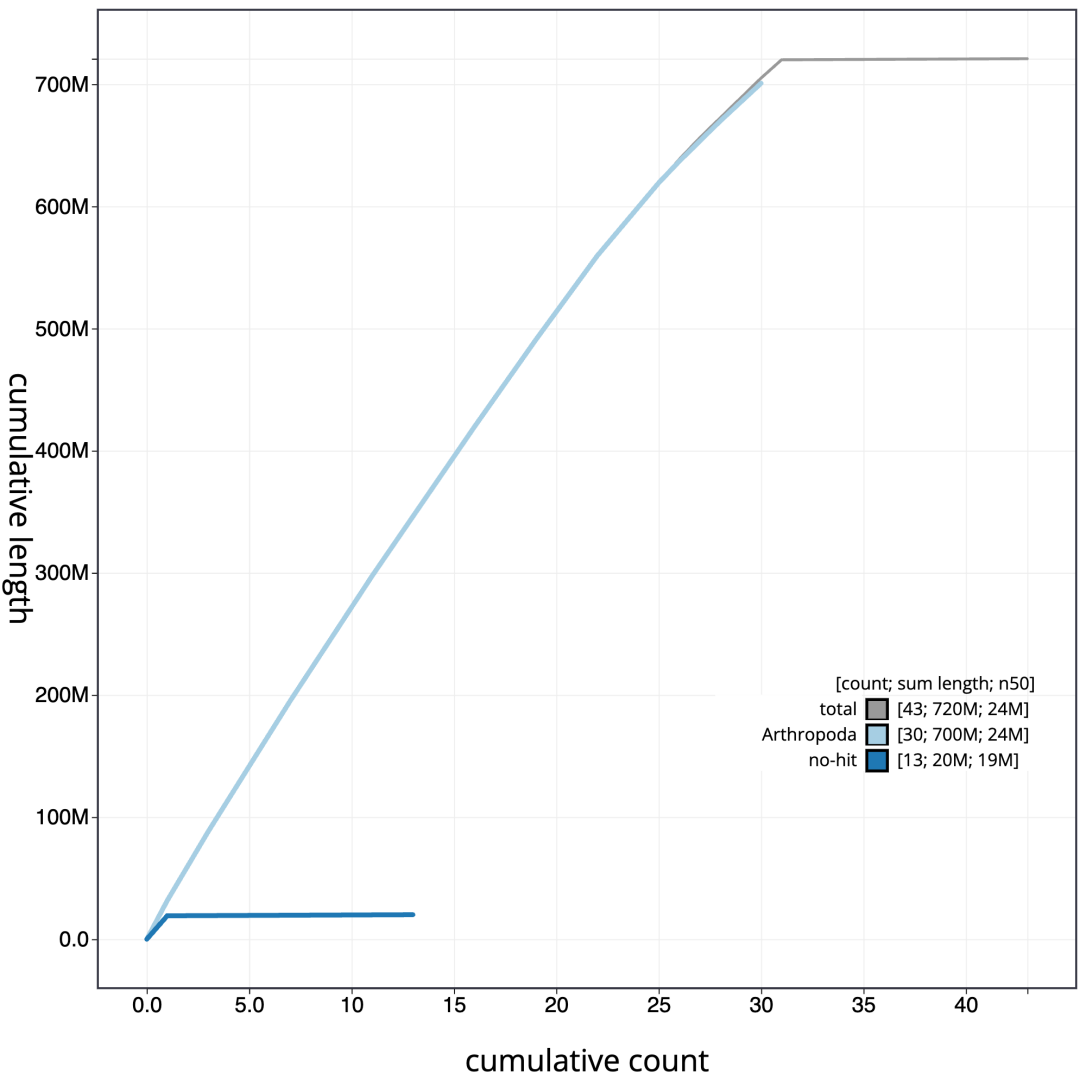
Genome assembly of *Conistra vaccinii*, ilConVacc3.1: BlobToolKit cumulative sequence plot. The grey line shows cumulative length for all scaffolds. Coloured lines show cumulative lengths of scaffolds assigned to each phylum using the buscogenes taxrule. An interactive version of this figure is available at https://blobtoolkit.genomehubs.org/view/Conistra%20vaccinii/dataset/CANUHQ01/cumulative.

**Figure 5.**
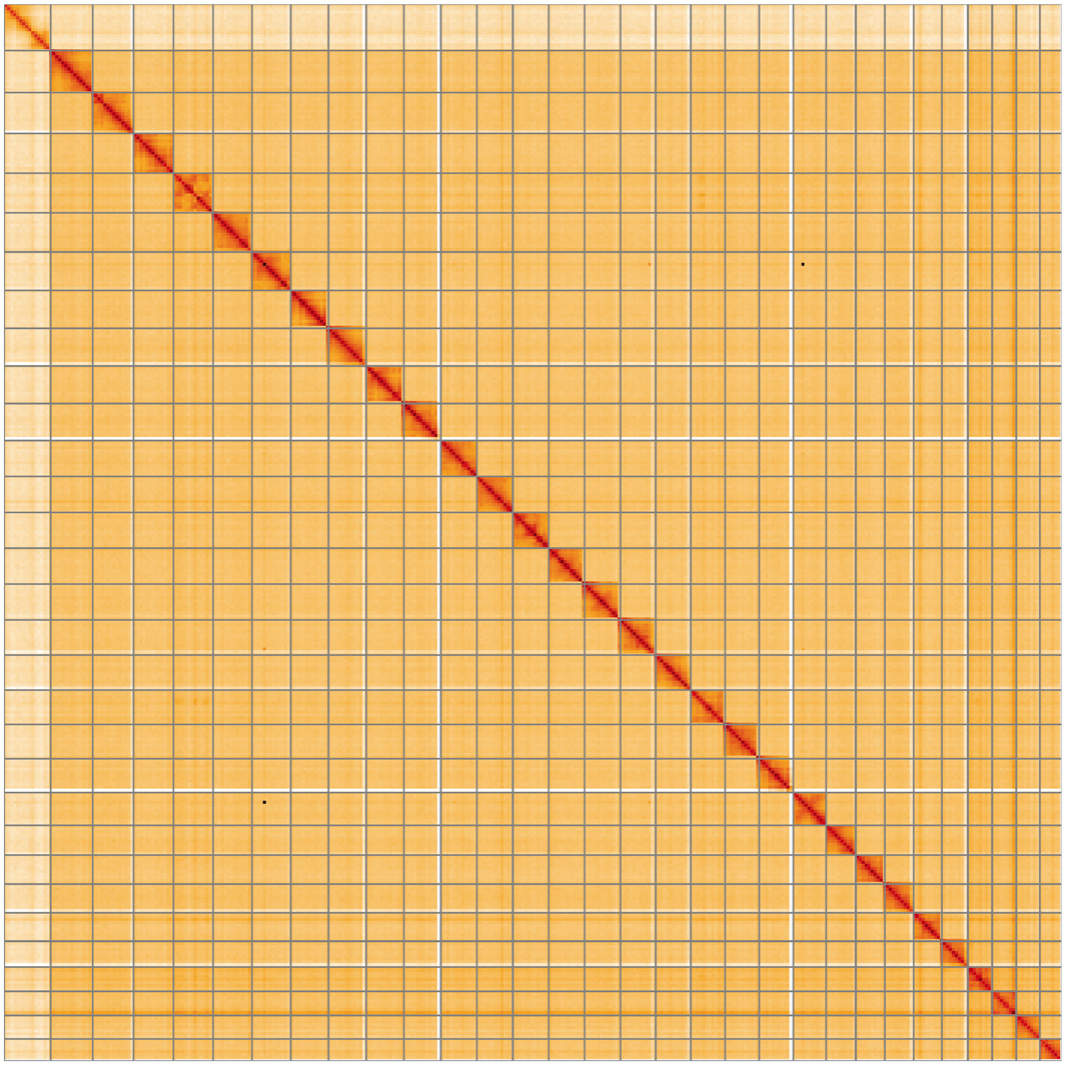
Genome assembly of *Conistra vaccinii*, ilConVacc3.1: Hi-C contact map of the ilConVacc3.1 assembly, visualised using HiGlass. Chromosomes are shown in order of size from left to right and top to bottom. An interactive version of this figure may be viewed at https://genome-note-higlass.tol.sanger.ac.uk/l/?d=eDBoITIdQUW5SuXhgYE6hw.

The final assembly has a total length of 720.8 Mb in 42 sequence scaffolds with a scaffold N50 of 24.5 Mb ( Table 1). The snailplot in Figure 2 provides a summary of the assembly statistics, while the distribution of assembly scaffolds on GC proportion and coverage is shown in Figure 3. The cumulative assembly plot in Figure 4 shows curves for subsets of scaffolds assigned to different phyla. Most (99.92%) of the assembly sequence was assigned to 31 chromosomal-level scaffolds, representing 30 autosomes and the Z sex chromosome. Chromosome-scale scaffolds confirmed by the Hi-C data are named in order of size ( Figure 5; Table 2). While not fully phased, the assembly deposited is of one haplotype. Contigs corresponding to the second haplotype have also been deposited. The mitochondrial genome was also assembled and can be found as a contig within the multifasta file of the genome submission.

**Table 1.** Genome data for *Conistra vaccinii*, ilConVacc3.1.

Project accession data		
Assembly identifier	ilConVacc3.1	
Assembly release date	2023-01-23	
Species	*Conistra vaccinii*	
Specimen	ilConVacc3	
NCBI taxonomy ID	706635	
BioProject	PRJEB57660	
BioSample ID	SAMEA9359439	
Isolate information	ilConVacc3: thorax (DNA sequencing) ilConVacc1: head and thorax (Hi-C data)	
Assembly metrics *		*Benchmark*
Consensus quality (QV)	68	*≥ 50*
*k*-mer completeness	100%	*≥ 95%*
BUSCO **	C:99.1%[S:98.3%,D:0.8%],F:0.2%,M:0.8%,n:5,286	*C ≥ 95%*
Percentage of assembly mapped to chromosomes	99.92%	*≥ 95%*
Sex chromosomes	Z chromosome	*localised homologous pairs*
Organelles	Mitochondrial genome assembled	*complete single alleles*
Raw data accessions		
PacificBiosciences SEQUEL II	ERR10499352	
Hi-C Illumina	ERR10501000, ERR10501006	
PolyA RNA-Seq Illumina	ERR10501005	
Genome assembly		
Assembly accession	GCA_948150665.1	
*Accession of alternate haplotype*	GCA_948150745.1	
Span (Mb)	720.8	
Number of contigs	99	
Contig N50 length (Mb)	16.0	
Number of scaffolds	42	
Scaffold N50 length (Mb)	24.5	
Longest scaffold (Mb)	31.6	
Genome annotation		
Number of protein-coding genes	13,109	
Number of non-coding genes	1,867	
Number of gene transcripts	22,888	

* Assembly metric benchmarks are adapted from column VGP-2020 of “Table 1: Proposed standards and metrics for defining genome assembly quality” from ( Rhie *et al.*, 2021). ** BUSCO scores based on the lepidoptera_odb10 BUSCO set using v5.3.2. C = complete [S = single copy, D = duplicated], F = fragmented, M = missing, n = number of orthologues in comparison. A full set of BUSCO scores is available at https://blobtoolkit.genomehubs.org/view/Conistra%20vaccinii/dataset/CANUHQ01/busco.

**Table 2.** Chromosomal pseudomolecules in the genome assembly of Conistra vaccinii, ilConVacc3.

INSDC accession	Chromosome	Length (Mb)	GC%
OX411316.1	1	28.59	38.0
OX411317.1	2	27.9	38.0
OX411318.1	3	27.09	37.5
OX411319.1	4	27.0	38.0
OX411320.1	5	26.61	38.0
OX411321.1	6	26.19	37.5
OX411323.1	8	25.76	38.0
OX411322.1	7	25.76	38.0
OX411324.1	9	25.62	38.0
OX411325.1	10	25.14	38.0
OX411327.1	12	24.48	38.0
OX411326.1	11	24.48	38.0
OX411328.1	13	24.46	37.5
OX411329.1	14	24.39	38.0
OX411330.1	15	24.36	38.0
OX411331.1	16	23.97	38.0
OX411332.1	17	23.87	38.0
OX411333.1	18	23.48	38.5
OX411334.1	19	23.26	38.0
OX411335.1	20	23.15	38.0
OX411336.1	21	22.33	38.5
OX411337.1	22	20.15	38.0
OX411338.1	23	19.89	38.0
OX411339.1	24	19.64	38.5
OX411340.1	25	19.2	38.5
OX411341.1	26	17.6	38.0
OX411342.1	27	16.56	39.5
OX411343.1	28	16.41	38.5
OX411344.1	29	16.02	39.0
OX411345.1	30	14.98	38.5
OX411315.1	Z	31.63	38.0
OX411346.1	MT	0.02	19.5

The estimated Quality Value (QV) of the final assembly is 68 with *k*-mer completeness of 100%, and the assembly has a BUSCO v5.3.2 completeness of 99.1% (single = 98.3%, duplicated = 0.8%), using the lepidoptera_odb10 reference set ( *n* = 5,286).

Metadata for specimens, barcode results, spectra estimates, sequencing runs, contaminants and pre-curation assembly statistics are given at https://links.tol.sanger.ac.uk/species/706635.

## Genome annotation report

The *Conistra vaccinii* genome assembly (GCA_948150665.1) was annotated using the Ensembl rapid annotation pipeline ( Table 1; https://rapid.ensembl.org/Conistra_vaccinii_GCA_948150665.1/Info/Index). The resulting annotation includes 22,888 transcribed mRNAs from 13,109 protein-coding and 1,867 non-coding genes.

## Methods

### Sample acquisition and nucleic acid extraction

A male *Conistra vaccinii* (specimen ID NHMUK013267905, ToLID ilConVacc3) was caught in a light trap in High Wycombe, Buckinghamshire, UK (latitude 51.63, longitude –0.74) on 2021-02-16. The specimen was collected and identified by David Lees (Natural History Museum) and then dry frozen at –80 °C.

The specimen used for Hi-C data and RNA sequencing (specimen ID Ox000322, ToLID ilConVacc1), was collected from Wytham Woods, Oxfordshire, UK (latitude 51.77, longitude –1.33) on 2020-01-08. The specimen was collected and identified by Liam Crowley (University of Oxford), and then frozen on dry ice.

High molecular weight (HMW) DNA was extracted at the Tree of Life laboratory, Wellcome Sanger Institute (WSI), using the main processes: sample preparation; sample homogenisation; HMW DNA extraction; HMW DNA fragmentation; and fragmented DNA clean-up. The ilConVacc3 sample was weighed and dissected on dry ice with tissue set aside for Hi-C sequencing (as per the protocol at https://dx.doi.org/10.17504/protocols.io.x54v9prmqg3e/v1). For sample homogenisation, thorax tissue was cryogenically disrupted using the Sample Homogenisation: Covaris cryoPREP® Automated Dry Pulverizer protocol ( https://dx.doi.org/10.17504/protocols.io.eq2lyjp5qlx9/v1), in which the sample is ground to a fine powder using a Covaris cryoPREP Automated Dry Pulveriser, receiving multiple impacts. HMW DNA was extracted by means of the HMW DNA Extraction: Automated MagAttract protocol ( https://dx.doi.org/10.17504/protocols.io.kxygx3y4dg8j/v1). HMW DNA was sheared into an average fragment size of 12–20 kb in a Megaruptor 3 system with speed setting 30, following the HMW DNA Fragmentation: Diagenode Megaruptor®3 for PacBio HiFi ( https://dx.doi.org/10.17504/protocols.io.8epv5x2zjg1b/v1). Sheared DNA was purified following either the Manual solid-phase reversible immobilisation (SPRI) protocol ( https://dx.doi.org/10.17504/protocols.io.kxygx3y1dg8j/v1) or the Automated SPRI protocol for ( https://dx.doi.org/10.17504/protocols.io.q26g7p1wkgwz/v1) for higher throughput. In brief, the method uses a 1.8X ratio of AMPure PB beads to sample to eliminate shorter fragments and concentrate the DNA. The concentration of the sheared and purified DNA was assessed using a Nanodrop spectrophotometer and Qubit Fluorometer and Qubit dsDNA High Sensitivity Assay kit. Fragment size distribution was evaluated by running the sample on the FemtoPulse system.

RNA was extracted from abdomen tissue of ilConVacc1 in the Tree of Life Laboratory using the Life RNA Extraction: Automated MagMax™ *mir*Vana protocol ( https://dx.doi.org/10.17504/protocols.io.6qpvr36n3vmk/v1). The RNA concentration was assessed using a Nanodrop spectrophotometer and Qubit Fluorometer using the Qubit RNA Broad-Range (BR) Assay kit. Analysis of the integrity of the RNA was done using the Agilent RNA 6000 Pico Kit and Eukaryotic Total RNA assay.

Protocols employed by the Tree of Life laboratory are publicly available on protocols.io: https://dx.doi.org/10.17504/protocols.io.8epv5xxy6g1b/v1.

### Sequencing

Pacific Biosciences HiFi circular consensus DNA sequencing libraries were constructed according to the manufacturers’ instructions. Poly(A) RNA-Seq libraries were constructed using the NEB Ultra II RNA Library Prep kit. DNA and RNA sequencing was performed by the Scientific Operations core at the WSI on Pacific Biosciences SEQUEL II (HiFi), Illumina HiSeq 4000 (RNA-Seq) and Illumina NovaSeq 6000 (10X) instruments. Hi-C data were also generated from head and thorax tissue of ilConVacc1 using the Arima2 kit and sequenced on the Illumina NovaSeq 6000, Illumina NovaSeq 6000 instrument.

### Genome assembly, curation and evaluation

Assembly was carried out with Hifiasm ( Cheng *et al.*, 2021) and haplotypic duplication was identified and removed with purge_dups ( Guan *et al.*, 2020). The assembly was then scaffolded with Hi-C data ( Rao *et al.*, 2014) using YaHS ( Zhou *et al.*, 2023). The assembly was checked for contamination and corrected as described previously ( Howe *et al.*, 2021). Manual curation was performed using HiGlass ( Kerpedjiev *et al.*, 2018) and Pretext ( Harry, 2022). The mitochondrial genome was assembled using MitoHiFi ( Uliano-Silva *et al.*, 2023), which runs MitoFinder ( Allio *et al.*, 2020) or MITOS ( Bernt *et al.*, 2013) and uses these annotations to select the final mitochondrial contig and to ensure the general quality of the sequence.

A Hi-C map for the final assembly was produced using bwa-mem2 ( Vasimuddin *et al.*, 2019) in the Cooler file format ( Abdennur & Mirny, 2020). To assess the assembly metrics, the *k*-mer completeness and QV consensus quality values were calculated in Merqury ( Rhie *et al.*, 2020). This work was done using Nextflow ( Di Tommaso *et al.*, 2017) DSL2 pipelines “sanger-tol/readmapping” ( Surana *et al.*, 2023a) and “sanger-tol/genomenote” ( Surana *et al.*, 2023b). The genome was analysed within the BlobToolKit environment ( Challis *et al.*, 2020) and BUSCO scores ( Manni *et al.*, 2021; Simão *et al.*, 2015) were calculated.

Table 3 contains a list of relevant software tool versions and sources.

**Table 3.** Software tools: versions and sources.

Software tool	Version	Source
BlobToolKit	4.1.7	https://github.com/blobtoolkit/blobtoolkit
BUSCO	5.3.2	https://gitlab.com/ezlab/busco
Hifiasm	0.16.1-r375	https://github.com/chhylp123/hifiasm
HiGlass	1.11.6	https://github.com/higlass/higlass
Merqury	MerquryFK	https://github.com/thegenemyers/MERQURY.FK
MitoHiFi	2	https://github.com/marcelauliano/MitoHiFi
PretextView	0.2	https://github.com/wtsi-hpag/PretextView
purge_dups	1.2.3	https://github.com/dfguan/purge_dups
sanger-tol/genomenote	v1.0	https://github.com/sanger-tol/genomenote
sanger-tol/readmapping	1.1.0	https://github.com/sanger-tol/readmapping/tree/1.1.0
YaHS	1.1a2	https://github.com/c-zhou/yahs

### Genome annotation

The Ensembl gene annotation system ( Aken *et al.*, 2016) was used to generate annotation for the *Conistra vaccinii* assembly (GCA_948150665.1). Annotation was created primarily through alignment of transcriptomic data to the genome, with gap filling via protein-to-genome alignments of a select set of proteins from UniProt ( UniProt Consortium, 2019).

### Wellcome Sanger Institute – Legal and Governance

The materials that have contributed to this genome note have been supplied by a Darwin Tree of Life Partner. The submission of materials by a Darwin Tree of Life Partner is subject to the **‘Darwin Tree of Life Project Sampling Code of Practice’**, which can be found in full on the Darwin Tree of Life website here. By agreeing with and signing up to the Sampling Code of Practice, the Darwin Tree of Life Partner agrees they will meet the legal and ethical requirements and standards set out within this document in respect of all samples acquired for, and supplied to, the Darwin Tree of Life Project.

Further, the Wellcome Sanger Institute employs a process whereby due diligence is carried out proportionate to the nature of the materials themselves, and the circumstances under which they have been/are to be collected and provided for use. The purpose of this is to address and mitigate any potential legal and/or ethical implications of receipt and use of the materials as part of the research project, and to ensure that in doing so we align with best practice wherever possible. The overarching areas of consideration are:

•   Ethical review of provenance and sourcing of the material

•   Legality of collection, transfer and use (national and international)

Each transfer of samples is further undertaken according to a Research Collaboration Agreement or Material Transfer Agreement entered into by the Darwin Tree of Life Partner, Genome Research Limited (operating as the Wellcome Sanger Institute), and in some circumstances other Darwin Tree of Life collaborators.

## Data Availability

European Nucleotide Archive: *Conistra vaccinii* (the chestnut). Accession number PRJEB57660; https://identifiers.org/ena.embl/PRJEB57660 ( Wellcome Sanger Institute, 2023). The genome sequence is released openly for reuse. The *Conistra vaccinii* genome sequencing initiative is part of the Darwin Tree of Life (DToL) project. All raw sequence data and the assembly have been deposited in INSDC databases. Raw data and assembly accession identifiers are reported in Table 1.

## Author information

Members of the Natural History Museum Genome Acquisition Lab are listed here: https://doi.org/10.5281/zenodo.7139035.

Members of the University of Oxford and Wytham Woods Genome Acquisition Lab are listed here: https://doi.org/10.5281/zenodo.7125292.

Members of the Darwin Tree of Life Barcoding collective are listed here: https://doi.org/10.5281/zenodo.4893703.

Members of the Wellcome Sanger Institute Tree of Life programme are listed here: https://doi.org/10.5281/zenodo.4783585.

Members of Wellcome Sanger Institute Scientific Operations: DNA Pipelines collective are listed here: https://doi.org/10.5281/zenodo.4790455.

Members of the Tree of Life Core Informatics collective are listed here: https://doi.org/10.5281/zenodo.5013541.

Members of the Darwin Tree of Life Consortium are listed here: https://doi.org/10.5281/zenodo.4783558.
