# Supplementary material for: The genome sequence of the Chestnut, Conistra vaccinii (Linnaeus, 1761)
Source: Wellcome Open Res. 2023 Nov 17;8:532. [Version 1] doi: 10.12688/wellcomeopenres.20346.1 (PMC11021882; doi:10.12688/wellcomeopenres.20346.1)
Supplement: Peer review history

# Reviewer report: Reviewer response for version 1

**DOI:** 10.21956/wellcomeopenres.22530.r79001

**Published:** 2024-04-16

**Authors:** Annabel Whibley

**Affiliations:** 1 Bragato Research Institute, Lincoln, New Zealand; 2 The University of Auckland, Auckland, Auckland, New Zealand

Lees and colleagues report the high-quality genome assembly and annotation of the Chestnut, a common noctuid moth in the British Isles. The natural history section gives a concise summary of the organism and notes interesting features of the biology and taxonomy of the chestnut.  

 The assembly and report follow carefully developed DToL workflows and templates and these comprehensively capture metadata for software versions and assembly properties.

 I have just minor questions/comments.

1. Although this is stated in the methods, please also highlight in the main text (in the genome sequence report section) that the HiC/RNA seq data comes from a second individual.
2. Can I query the bead ratio stated in the methods *“In brief, the method uses a 1.8X ratio of AMPure PB beads to sample to eliminate shorter fragments and concentrate the DNA.”*  In my understanding, and from the guidelines on protocol.io, to eliminate smaller fragments, a lower ratio of beads to sample would be used. Should this be 0.6x?

Are sufficient details of methods and materials provided to allow replication by others?

Yes

Is the rationale for creating the dataset(s) clearly described?

Yes

Are the datasets clearly presented in a useable and accessible format?

Yes

Are the protocols appropriate and is the work technically sound?

Yes

Reviewer Expertise:

Genomics, bioinformatics, evolution

I confirm that I have read this submission and believe that I have an appropriate level of expertise to confirm that it is of an acceptable scientific standard.

# Reviewer report: Reviewer response for version 1

**DOI:** 10.21956/wellcomeopenres.22530.r71187

**Published:** 2024-01-31

**Authors:** Guanghong Liang

**Affiliations:** 1 Fujian Agriculture and Forestry University, Fuzhou, Fujian, China

This paper has sequenced and assembled the genome of *Conistra vaccinii* which is reported to be 720.8 megabytes in size, comprising 31 chromosomes, and also completed the assembly of a 15.44 kilobase mitochondrial genome, and elaborated in detail the entire process from genome sequencing to assembly, revealed a substantial number of protein-coding genes within the *Conistra vaccinii* genome, which will be very helpful to identify this species from numerous specimen by using light trap, and also play a significant role in reveal phylogenetic relationships in Noctuid moths.

 However, the paper also needs improvement as follow:

1. The sequencing depth needs to be discussed.
2. The abstract section is overly succinct, I would suggest that the abstract should be rewritten  to increase its appeal.
3. The significance of objective in the introduction is also insufficient, for example, why did author try to reveal the genome sequence of *Conistra vaccinii* , just for interesting? is there any taxonomy trouble to  identify those specimen by using light trap?
4. The clarity of the images is inadequate, especially two specimen features are incomplete.
5. Please add the detail information about instruments and reagents used in this work, as well as the names and countries of the manufacturers in the Materials and Methods section.

Are sufficient details of methods and materials provided to allow replication by others?

Yes

Is the rationale for creating the dataset(s) clearly described?

Yes

Are the datasets clearly presented in a useable and accessible format?

Yes

Are the protocols appropriate and is the work technically sound?

Yes

Reviewer Expertise:

Entomology

I confirm that I have read this submission and believe that I have an appropriate level of expertise to confirm that it is of an acceptable scientific standard.
